# Self-Perceived Health, Objective Health, and Quality of Life among People Aged 50 and Over: Interrelationship among Health Indicators in Italy, Spain, and Greece

**DOI:** 10.3390/ijerph17072414

**Published:** 2020-04-02

**Authors:** Laura Maniscalco, Silvana Miceli, Filippa Bono, Domenica Matranga

**Affiliations:** 1 Department of Biomedicine, Neuroscience and Advanced Diagnostics, University of Palermo, Palermo 90127, Italy; laura.maniscalco04@unipa.it; 2 Department of Psychology, Educational Science and Human Movement, University of Palermo, Palermo 90128, Italy; silvana.miceli56@unipa.it; 3 Department of Economics, Business and Statistics (SEAS), University of Palermo, Palermo 90128, Italy; filippa.bono@unipa.it; 4 Department of Health Promotion, Mother and Infant Care, Internal and Specialized Medicine “G. D’Alessandro”, University of Palermo, Palermo 90127, Italy

**Keywords:** additive Bayesian network, self-perceived health, quality of life, chronic diseases cognitive measures

## Abstract

It is well known that self-perceived health (SPH), even if it is a subjective health indicator, is significantly associated with objective health and quality of life (QoL) in the general population. Whether it can be considered an indicator of cognitive functioning and quality of life in the elderly is still an open issue. This study used a data-driven approach to investigate the interrelationship among SPH, non-communicable diseases (NCDs), QoL, and cognitive functioning to answer this question. The study sample included information about 12,831 people living in Italy, Spain, and Greece, extracted from the Survey on Health, Aging, and Retirement in Europe, in the year 2015. The additive Bayesian networks methodology was used to identify the best directed acyclic graphs (DAG) for SPH, QoL, and NCDs. Results were given as posterior estimates of generalized linear models (GLM) coefficients, with 95% credibility intervals. Good SPH was associated with a decreasing number of chronic diseases in Italy (coeff = −0.52, 95%CI: [−0.59, −0.44]), Spain (coeff = −0.53, 95%CI: [−0.60, −0.46]) and Greece (coeff = −0.57, 95%CI: [−0.64, −0.50]). Age and Body Mass Index were determinants of NCDs in all countries. QoL of elderly was associated with SPH in Italy (coeff = 0.12, 95%CI: [0.10, 0.14]), Spain (coeff = 0.16, 95%CI: [0.15, 0.18]), and Greece (coeff = 0.18, 95%CI: [0.16, 0.20]). The number of NCDs was higher for people who were not employed in Spain (coeff = 0.45, 95%CI: [0.37, 0.53]) and was decreasing for a unitary increase in years of education in Greece (coeff = −0.12, 95%CI: [−0.14, −0.09]). As a general rule, the framework of the interrelationship among NCDs, SPH, and QoL was similar for Italy, Spain, and Greece. The connections found among indicators could be proposed to identify strategies for health promotion and healthy aging among people aged 50 and above, which are viable in general and at a country level. Reinforcing strategies targeted at some health indicators could have relevant effects on other related indicators.

## 1. Introduction

In the world, the population aged 60 years and over exceeded nine hundred million in 2017, and it is expected to reach nearly 2.1 billion in 2050 [1]. Population aging is a global phenomenon and a priority of health policy for all countries since elderly people represent a burden for the active population both in terms of healthcare expenditure and caregiving. Some statistics show that a 65-year-old individual with a serious chronic illness spends between $1000 to $2000 more per year in health care services than a similar adult without such a condition [2]. As a consequence, detecting health determinants, identifying actions to postpone the onset of disease, and ensuring healthy aging [3] are strategic issues. Health promotion targeted at elderly people, to keep cognitive functions unaltered and prevent noncommunicable diseases, must be extended to middle-aged people too, as virtuous behaviors and correct lifestyle affecting health in later life must be established earlier in adulthood. Therefore, proposing a comprehensive system of health indicators, which are relevant for elderly and middle-aged people, can be considered a relevant methodologic matter for health policy. This study aimed to identify the interrelationships among health indicators in this specific population. To this end, data were extracted from the Survey on Health, Ageing and Retirement in Europe (SHARE) [4], which is a multidisciplinary and cross-national panel database on health, socio-economic status (SES), social and family networks of people aged 50 or above, living in twenty-seven European countries and Israel [5]. Self-perceived health (SPH), quality of life (QoL) in older ages, chronic or non-communicable diseases (NCDs), global activity limitation, lifestyle, and cognitive functioning are some of the statistical indicators used to capture health multidimensionality of elderly people. It has already been established that SPH supplies information in line with the objective health status [6,7] and quality of life [8] in the general population and that chronic conditions, lifestyle factors, and cognitive functioning are significantly associated with health-related quality of life of adults [9]. Whether SPH can be considered a broad indicator of cognitive functioning and QoL in the elderly is still debated.

SPH measures “subjective health”, as it synthesizes all the information regarding an individual’s health, such as physical functioning in everyday life, lifestyle conditions, specific disease characteristics (severity and prognosis), and cultural background [10]. The main determinants of SPH are socio-economic factors [6,11] as well as biological, physiological, and psychosocial factors. The World Health Organization (WHO) and the European Commission [12] recommend the use of SPH for health monitoring, and several authors suggest that SPH correlates well with objective health status, in the general population [7], in specific groups [13], and in the older population [14]. SPH is also a comprehensive indicator of lifestyle-related health status because it is significantly associated with some of the lifestyle habits, such as weight change, exercise, smoking, and rest [15].

QoL can be defined according to either a subjective or an objective vision [16]. Following the subjective approach, QoL is based on human needs, emotional wellbeing, and expectations [17]. It is defined as “a conscious cognitive judgment of satisfaction with one’s life” [18] or “an individual’s perception of their position in life in the context of the culture and value systems in which they live and in relation to their goals, expectations, standards, and concerns” [19]. According to the objective approach [20], QoL is defined as “an overall well-being that comprises objective and subjective evaluations of physical, material, social, and emotional well-being together with the extent of personal development and purposeful activity, all weighted by a personal set of values” [21]. The health status of an individual, with his/her functional abilities and clinical symptoms, together with his/her health perception represents one dimension of QoL [16].

The objective health status of an individual is defined by his/her current or past diseases, both acute and chronic, and clinical parameters [13]. To attain a broad measurement of the objective health for elderly people, NCDs, the lifestyle, global activity limitation, and cognitive functioning should be included as statistical indicators. The global activity limitations indicator is a measure of the functional status of subjects, which corresponds to the occurrence of long-term limitations in usual activities due to a health problem [22]. This indicator is widely recognized as a powerful determinant of SPH in the elderly [23] and represents a key element in the implementation of targeted health interventions. In the same way, the evaluation of cognitive functions represents a very important element in determining the health status of the elderly. In fact, it is through cognitive functions that people perform most of the daily actions, even the simplest ones, such as driving a car or doing the shopping, and maintaining social relationships and social activities. There is abundant literature showing that cognitive functions can be enhanced and maintained through protective factors, which are linked to lifestyle [24,25,26] and quality of life [27].

To make it more intriguing to disentangle factors among health status, health perception, and QoL, the abundant literature showing the individual’s socio-economic status as a common determinant must also be considered. It has been shown that lifestyle and socio-economic status (SES) indicators are determinants for SPH [8], QoL in older ages [28], and cognitive functioning [27,29].

On the whole, the first contribution of this study was evaluating the different indicators in an integrated way to show whether SPH can be considered a correlate of cognitive functioning and QoL of people aged 50 and over. The second important contribution of this study was focusing on Italy, Spain, and Greece, which are three Mediterranean European Union (EU) member states that share a similar profile from demographic, health, and care points of view. These three countries enjoy the same fragmented system of welfare provision, with income guarantees linked to work position, a high percentage of social expenditure financed through contributions, and a strong reliance on family and on the charitable sector [30]. In Italy, Spain, and Greece, welfare has also undergone major transformations over the past few years. In fact, between 2009 and 2010, Greece experienced a serious financial crisis with the highest deficit rate in the EU, which led to a tightening of fiscal policy, mainly centered on cuts to welfare spending. In the years immediately following, Italy and Spain also underwent important austerity policies that inevitably produced significant effects on the retirement system, on labor market policies, but above all on health policies. This has created significant problems for the maintenance of social protection systems in Southern Europe. There is, therefore, an evident need to monitor the trend of indicators highlighted in our study (NCDs, SPH, and QoL) in the three different geographical contexts, to implement targeted interventions in the field of public health research and practice.

## 2. Data and Methods

Data for this study was extracted from the sixth (year = 2015) wave of the SHARE Survey.

Data collection was executed through computer-assisted personal interviews, which were conducted face-to-face with the target respondent and willing partners or spouses. For people affected by cognitive limitations, including Alzheimer’s disease, dementia, and senility, information was obtained through a proxy interview. Some interviews were supplemented with the participant’s completion of a written questionnaire, which included additional questions on subjects such as mental and physical health, health care, and social networks.

For the purposes of the current investigation, only the information concerning people from Spain, Italy, and Greece, was extracted. Only normal aging was considered, so people with Alzheimer’s disease, dementia, and senility were excluded. Therefore, the study sample consisted of 12,831 people, of which 4110 were Italian (32%), 4308 were Spanish (34%), and 4413 were Greek (34%). Data are available free of charge for registered users at http://www.share-project.org and are distributed through twenty-five regular and four special module files.

### 2.1. Variables

To measure SPH, the single-item question “In general, would you say your health is…” was asked, with ordered response categories on a five-point scale from excellent to poor.

QoL in older ages was measured through the 12-item version of the CASP scale (CASP-12), which is the reduced version of the 19-item CASP scale and measures the four dimensions of needs Control, Autonomy, Self-realization, and Pleasure. The questions involved a list of statements used to describe people’s lives or how people feel and what people think, with answers coded on a 4-point scale indicating how often (1. Often, 2. Sometimes, 3. Rarely, and 4. Never), if at all, the interviewed person had experienced these feelings and thoughts. All items were recorded in such a way that higher scores indicate a higher level of QoL. CASP-12 ranged between 12 and 48 [31,32].

The number of NCDs and the global activity limitation index were included as measures of objective health status. The global activity limitation index corresponded to the question: “For at least the past 6 months, to what extent have you been limited because of a health problem in activities people usually do? Would you say you have been …” severely limited/limited but not severely or/not limited at all? For the purposes of the study, answers were categorized as “not limited” vs. “limited”. Cognitive functioning was assessed through two memory tests and one executive function. For the memory tests, we used the immediate (ITest) and delayed (DTest) verbal recall test from the Ten-Word Delayed Recall Test, both ranging between 0 and 10 [26]. To examine executive function, we used the score of a semantic verbal fluency (FTest) test consisting of asking the participant to name as many animals as possible correctly during a one-minute period. This score ranged between 0 and 100. In this type of task, in addition to the linguistic component, there is “fluency” that is based on non-linguistic skills, such as selective attention and rapidity, which are necessary to guide the search and recovery of elements in the semantic memory [33]. The correct execution of the test assumes the integrity of the working memory, too [34]. Gender, age, living in a couple, and country of residence were included as demographic variables, while years of education, current job status, household total net income, and household net worth were chosen as SES variables. To measure lifestyle, we included Body Mass Index (BMI), smoking status (“Yes” vs. “No”), and physical inactivity (“Yes” vs. “No”). Limited to multivariable statistical analysis, some variables were transformed into binary ones: age (“>65” vs. “<=65”), current job status (“Not employed”, which included homemakers/permanently sick/retired/unemployed vs. “Employed”, which included employed/self-employed/other), living in a couple (“Not in couple” vs. “In couple”).

### 2.2. Statistical Methods

Continuous variables were described by the following summary statistics: mean and standard deviation (the latter is reported in brackets), median, and range. Categorical variables were analyzed as counts and percentages. To assess the statistical significance of the difference among countries, the Chi-squared test was used for categorical variables, and the ANOVA test was used for continuous variables.

The interrelationships among SPH, the objective health status, QoL, cognitive tasks, and SES were modeled through additive Bayesian networks (ABNs), a class of probabilistic models that unify Bayesian networks and generalized linear models (GLM) [35]. A Bayesian network (BN) uses an acyclic directed graph (DAG) to represent a set of random variables as nodes and their conditional dependencies as arrows between the nodes. A node is called an ancestor if it influences another variable, which, in turn, assumes the role of the descendant. The DAG is called acyclic because a node could not be its own ancestor or its own descendent. A BN usually models the joint distribution of a set of random variables through a multivariate continuous or discrete distribution. These assumptions can be too restrictive for real data analysis and more flexible graphical models are necessary for mixed (continuous and discrete) random variables. Recently, Pittavino et al. [36] proposed ABNs as an extension of BNs by allowing each random variable to be modeled as an exponential distribution through a generalized linear model [30]. The ABN considers all the variables jointly and aims to identify all direct and indirect relationships between them. An edge between two variables in the ABN model represents a “direct” relationship, whereas an “indirect” relationship is defined as a relationship between two variables through an intermediate variable. As the ABN is defined in a Bayesian context, each parameter, and the DAG as a whole, are defined as random variables. In this paper, binary variables were modeled through a binomial distribution and logit link, quantitative variables through a normal distribution with identity link, while count variables were modeled through a Poisson distribution with a log link. A uniform prior was given to the DAG structure, uninformative Gaussian priors were applied for the parameters at each node, and diffuse Gamma distributions with shape and scale of 0.001 were used for the precision.

To identify the best DAG, an exact search method was used with the log marginal likelihood as the goodness of fit metric [35]. An exact search is based on the increase in the maximum number of parents allowed per node (the number of covariates allowed in each model) until the goodness of fit remains constant. This iterative approach is usually applied to avoid the increase in computational time needed to search across the model space of DAGs with larger parent limits. In this paper, the model selection procedure considered from one to twelve possible parents per node. In the second step, the marginal posterior log odds ratio and its 95% credibility interval was estimated for each parameter from the posterior distribution, expressed by the optimal DAG identified at the first step. In line with other literature, the log marginal likelihood was not checked for overfitting because the study sample was of a large size [37]. Results of ABN analysis were expressed as posterior estimates of GLM coefficients, with 95% credibility intervals.

Data were analyzed using the R software (version 3.3.2), and the ABN methodology was implemented using the “abn” package [38] in the R environment. The “fitabn” function was used to estimate the parameters of the linked variables and to represent their relationships graphically. For the sake of clarity, the graphics resulting from the “abn” package were adapted to show the parents of each health outcome one by one. A *p*-value < 0.05 was considered statistically significant. In this study, the estimated ABNs reported banned edges directed towards gender and age, since it is not theoretically possible for these variables to be influenced by other covariates.

## 3. Results

### 3.1. Sample Description

The sample included 5695 males (44%) and 7136 females (56%). The distribution by gender was similar in the three countries, while age was on average significantly higher in Spain (68.3 (9.9)) compared to Greece (66.9 (10.0)) and Italy (65.7 (9.4)) (*p* < 0.001). The years of education were 8.8 (4.6), on average, with Greek people being significantly more educated than Spaniards and Italians. On average, people within the sample were overweight (the average BMI was 27.0 (4.2)), mostly physically inactive (84% of the sample), and living in a couple (75%). The highest percentage of current smokers was observed in Greece (47%), followed by Italy (39%), and then Spain (36%). Nearly half of the sample was retired (47%), with the highest percentage of retired people in Italy (49%) compared to Spain and Greece (both 46%).

With regards to health indicators, people were, in general, affected by more than one NCD (1.7 (1.5)) with a slight difference between Greece and Spain and a bit more difference in Italy (1.5 (1.5)). Of the three examined cognitive tasks, there was a lower value for the executive task for the Greeks (12.4 (5.1)) compared to the Italians and Spaniards; there was a lower value for both memory tasks for Spanish subjects (4.2 (1.8) for the ITest and 2.8 (1.9) for DTest) compared to the other two groups. Regarding QoL in older ages, the CASP-12 score was higher for Spanish subjects (35.5 (6.2)) compared to the Italians and Greeks. Thirty-seven percent of the whole sample perceived poor or fair health, varying from a minimum of 31% observed for Greeks to 38% and 40% of Italians and Spaniards, respectively. Thirty-four percent of the whole sample suffered from global activity limitations, with a minimum of 28% for Greece to 36% for Spain and 38% for Italy (Table 1). The correlation matrix between pairs of variables is given as Supplement materials.

### 3.2. Description of DAGs for Spain, Greece, and Italy

To find the best DAG, the maximum number of parents allowed for each node has to increase until the log marginal likelihood remains constant. The model selection procedure identified a maximum number of parents equal to six for all countries.

**Figure 1:**
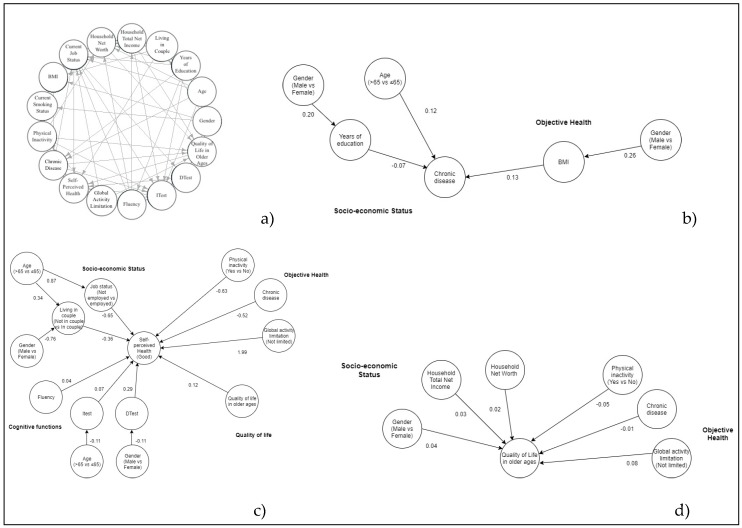
Additive Bayesian networks describing the interrelationship for all health indicators (**a**), chronic diseases (**b**), Self-perceived health (**c**), quality of life (QoL) (**d**) for Italian elderly. Source: The Survey on Health, Aging, and Retirement in Europe, wave 6th, 2015 year. BMI = Body Mass Index, Itest = Immediate verbal recall test, Dtest = Delayed verbal recall test.

**Figure 2:**
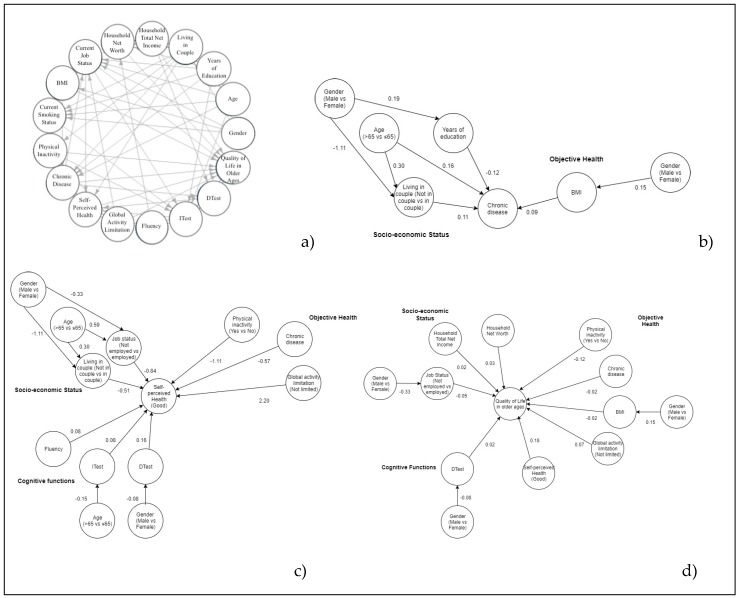
Additive Bayesian networks describing the interrelationship for all health indicators (**a**), chronic diseases (**b**), Self-perceived health (**c**), QoL (**d**) for Greek elderly. Source: The Survey on Health, Aging, and Retirement in Europe, wave 6th, 2015 year. BMI = Body Mass Index, Itest = Immediate verbal recall test, Dtest = Delayed verbal recall test.

**Figure 3:**
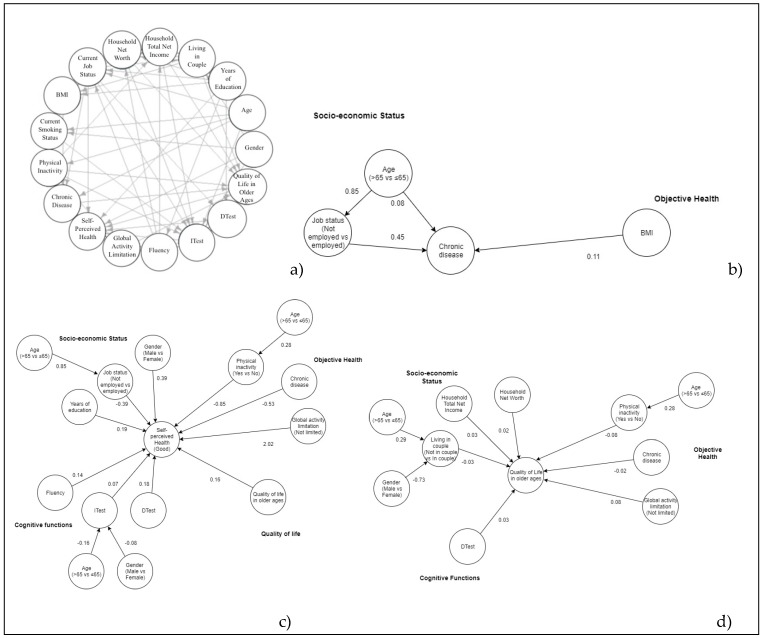
Additive Bayesian networks describing the interrelationship for all health indicators (**a**), chronic diseases (**b**), Self-perceived health (**c**), QoL (**d**) for Spanish elderly. Source: The Survey on Health, Aging, and Retirement in Europe, wave 6th, 2015 year. BMI = Body Mass Index, Itest = Immediate verbal recall test, Dtest = Delayed verbal recall test.

The final globally optimal DAGs, including all variables and one edge for every association, both direct and indirect, are shown, respectively, for Italian, Greek, and Spanish people (Figure 1a, Figure 2a and Figure 3a). For the sake of clarity, a graph of the different interrelationships with one health outcome at a time (NCDs, SPH, QoL) is presented for each country. Posterior estimates of GLM coefficients and respective 95% credibility intervals are shown in Table 2.

Relating the number of NCDs (Figure 1b, Figure 2b and Figure 3b), the optimal DAGs for Italian, Greek, and Spanish people show a direct association with BMI and age. For Spanish subjects, the number of NCDs is significantly higher for people not in employment. In Greek and Italian DAGs, the association with gender looks to be mediated by years of education and living in a couple (Greece only).

Relating to SPH (Figure 1c, Figure 2c and Figure 3c), the three DAGs show better-perceived health by decreasing the number of NCDs and by increasing the DTest. People without global activity limitations are more likely to enjoy better health, while those who are not in employment or are physically inactive are prone to worse health perception. Moreover, the Italian and Greek DAGs show people not living in a couple perceiving worse health than those living in a couple. SPH shows a direct association with QoL in the Italian and Spanish DAG. Males perceive better health than females in the Spanish DAG. The association with age seems to be mediated by current job status and ITest in all DAGs. Regarding gender, the indirect association with SPH passes through the ITest for Spanish DAGs and through the DTest and living in a couple for Greek and Italian DAGs.

In the Italian, Greek, and Spanish samples, QoL of elderly (Figure 1d, Figure 2d and Figure 3d) is better for people without global activity limitations and for people with higher household total net income and household net worth. QoL worsens for physically inactive people and by increasing the number of NCDs. Finally, in the Greek DAG, QoL shows a direct association with health perception.

## 4. Discussion

Through the discovery of interrelationship among SPH, objective health, and QoL, this study could demonstrate that SPH is significantly associated with cognitive functioning and QoL of people aged 50 and above, besides also confirming the well-known association with chronic diseases. In this way, knowledge can be pursued regarding what is actionable for health promotion and wellbeing improvement of elderly people in these Mediterranean countries. Maintaining and increasing functional capacity, maintaining or improving self-care, and fostering one’s social network, and also social participation and integration [27], contribute to a longer, more independent, and self-sufficient quality of life [39] with an important impact on individual health status. At the policy level, infrastructure investments can be suggested to favor active aging of elderly people, such as recreation centers with a supply of cultural programs, physical training, and other activities which stimulate high cognitive involvement to enhance executive functions.

These three Mediterranean countries were among those with the most pronounced pace of demographic change between 1974 and 2014. In 2015, these countries reported the highest peak in the proportion of elderly people in the population (between 18% and 21%), and fewer than 20% were living alone. They recorded the largest gender gaps in favor of men regarding the number of healthy life years at the age of 65. In addition, by combining the information on healthy life years with life expectancy, the biggest differences between males and females were found again in Spain and Greece, apart from Portugal and Cyprus [40].

As a general rule, the framework of this interrelationship was consistent in all three countries. The components of objective health and cognitive functions came out as determinants of SPH. In particular, the role of delayed recall was more remarkable compared to immediate recall and semantic verbal fluency, as it is the most sensitive among the measurements of cognitive impairment [41]. With regards to the components of objective health, many other studies showed a significant increase in the prevalence of all chronic diseases in association to lower health perception [6,7] and demonstrated that global activity limitation relates to SPH and mental and physical health problems [42].

In the current literature, the labor market, the educational system, and socio-demographic characteristics have been found as determinants of SPH in older age [8,43]. In addition, in our study, job status was found as a determinant of SPH in all three countries, but there was a significant association with years of education and gender only in Spain, with the major likelihood of perceiving good health for males compared to females [44]. Analogously, our finding confirms that a higher level of education is associated with better health perception among Spanish people aged 50 and above [45].

Regarding NCDs, age and BMI were found as determinants in all three countries. This result is in line with the causal trajectory of risk factors of NCDs, with four levels of causation from far away to closer to the disease, these being: physiological factors, lifestyle influences, environmental influences, and social structure. Age and BMI belong to the first and the fourth level, respectively [46]. Current job status was found to be associated with the number of NCDs in Spain, in agreement with other literature [47] Moreover, education is confirmed as a determinant both in Italy and Greece [48,49]. Not living in a couple was another risk factor for NCDs among elderly Greeks. It can be explained as lonely people are more prone to engage in unhealthier behaviors (smoking, low interest in health screening), to experience more psychological distress, and not have time to take care of themselves with physical activity [49].

For both SPH and NCDs, income did not play any role. This result is in line with other literature using the SHARE dataset, as it was found that both the number of NCDs and socioeconomic inequality in NCDs were more likely to be related to differences in education than income [50].

Another finding of the study was that people without any global activity limitation enjoyed better QoL in all three samples. QoL and health status are different concepts, as the first one is more associated with mental health, the second one more to physical functioning [51]. However, the current study showed that these constructs are correlated.

The relation between SPH and QoL can be twofold. In some cases, self-perceived health showed a direct relationship with quality of life. The way an elderly person perceives his/her own health condition steers his/her lifestyle and inevitably influences quality of life. In other cases, quality of life goes beyond the perceived state of health: for example, having good social relationships, being active and able to participate in socially significant activities can be very important for older people. In the three countries, our study showed a direct association between QoL and health perception. This result is in line with several other studies where QoL was associated with health improvement and the promotion of active aging [28]. Other studies have considered SPH as a direct measure of QoL, without considering other concepts, such as health or psychological wellbeing [52,53].

The study showed that the association with age for all health outcomes was not only direct but seemed to be mediated by cognitive measurements. This result, that should be confirmed with appropriate statistical methods, has important implications for the development of support practices related to the maintenance and enhancement of cognitive functions, considered as factors of protection from psychosocial risk. In fact, today, we tend to worry about our mental functioning when the decline is already evident, and we rarely take a preventive and empowering approach. Therefore, it becomes a priority to identify effective cognitive enhancement strategies that, by operating on the mechanisms of brain plasticity and contributing to the maintenance of a good cognitive reserve, are able to mediate the effects of aging.

The proposed methodology allowed us to study the associations among several variables simultaneously and to grasp the natural complexity of data more effectively than GLM. While GLM addresses the direct dependencies between risk factors and one single outcome, the ABN models the dependencies (both direct and indirect) among all the variables jointly. Compared to other statistical methods, the ABN focused on structure discovery with the aim of representing the core data generation process.

The main strength of our study was using a data-driven approach to infer the relationship among several health indicators and their determinants. Different studies have investigated the relationship among socioeconomic and lifestyle determinants with health outcomes one by one [31,54]. Few considered the relationship between pairs of health outcomes, such as SPH and objective health status [7], SPH and NCDs [6], NCDs and QoL [55], and SPH and QoL [8]. A recent study took into account NCDs, cognitive functioning, and lifestyle factors, but did not include SPH and global activity limitations [9].

There are, however, some limits to the present study. The first one regards some aspects of the variables used in this analysis. In fact, we could have obtained other results if we had used other QoL indicators, or different cognitive tasks from the three used in this paper. However, these tasks measure specific aspects of cognition, which deteriorate more with age, showing a constant decline both in healthy subjects and in subjects affected by Alzheimer’s disease [56]. Another limitation concerns the study design. As it is a cross-sectional study, it does not allow for testing of cause–effect relationships, but it helps us to formulate research hypotheses that could be the basis of subsequent confirmatory studies. Finally, another limitation regards the occurrence of selection bias, since the study subjects were the survivors of their cohorts following exclusion of people with cognitive impairments, and thus by definition the healthiest.

The connections found among different health dimensions could be relevant to identify intervention strategies that are viable in general and at a country level. It was demonstrated that acting to combat cognitive decline, to reduce the effects of global activity limitations in daily life, and to support a correct lifestyle to prevent the occurrence of NCDs should improve all health outcomes for middle-aged and elderly people. Furthermore, in line with the Health 2020 Agenda [57], the analysis of health indicators provides useful information for the design and implementation of health promotion strategies and health inequality reduction that take into account the specific characteristics of each country.

## Figures and Tables

**Table 1:** Descriptive statistics^§^ of 12,931 people aged 50 years and more living in Italy, Spain, and Greece.

	Greece	Italy	Spain	Total Sample	*p*
Age **					
Range	(50–95)	(50–102)	(50–102)	(50–102)	<0.0001
Median	66	65	67	66	
Mean(SD)	66.86 (9.96)	65.72 (9.41)	68.25 (10.06)	66.96 (9.87)	
Years of education **					
Range	(0–25)	(0–25)	(0–25)	(0–25)	<0.0001
Median	9	8	8	8	
Mean(SD)	9.51 (4.37)	8.59 (4.31)	8.33 (4.95)	8.82 (4.58)	
Household net income **					
Range	(0–47,170)	(0–47,040)	(0–47,000)	(0–47,170)	<0.0001
Median	10310	17580	14010	13620	
Mean(SD)	11,860 (9163.47)	18,560 (11,291.43)	15,540 (10,022.52)	15,250 (10,531.93)	
Household net worth **					
Range	(−169,200 520,700)	(−48,000 541,700)	(−94,350 543,300)	(−169,200 543,300)	<0.0001
Median	80,000	160,000	153,900	128,000	
Mean(SD)	105,000 (100,907.5)	178,600 (134,075.1)	174,300 (123,691.3)	151,800 (124,695.9)	
BMI^†^ **					
Range	(15.12 57.37)	(15.79 50.71)	(15.06 56.80)	(15.06 57.37)	<0.0001
Median	26.83	25.88	26.64	26.45	
Mean(SD)	27.36 (4.23)	26.35 (4.13)	27.10 (4.27)	26.95 (4.23)	
Gender *					N.S.
Female	2484 (56%)	2260 (55%)	2392 (56%)	7136 (56%)	
Male	1929 (44%)	1850 (45%)	1916 (44%)	5695 (44%)	
Living in couple *					<0.0001
In couple	3311 (75%)	3230 (79%)	3402 (79%)	9943 (78%)	
Not in couple	1102 (25%)	880 (21%)	906 (21%)	2888 (22%)	
Current job status *					<0.0001
Employed	1060 (24%)	1005 (24%)	961 (22%)	3026 (24%)	
Not employed	3353 (76%)	3105 (76%)	3347 (78%)	9805 (76%)	
Current smoking status *					0.047
No	2324 (53%)	2493 (61%)	2766 (64%)	7583 (59%)	
Yes	2089 (47%)	1617 (39%)	1542 (36%)	5248 (41%)	
Physical inactivity *					<0.0001
No	4079 (92%)	3129 (76%)	3606 (84%)	10814 (84%)	
Yes	334 (8%)	981 (24%)	702 (16%)	2017 (16%)	
NCDs **					<0.0001
Range	(0–12)	(0–9)	(0–9)	(0–12)	
Median	1	1	2	1	
Mean(SD)	1.70 (1.57)	1.49 (1.45)	1.77 (1.49)	1.66 (1.51)	
Fluency **					
Range	(0–77)	(0–70)	(0–93)	(0–93)	<0.0001
Median	12	15	15	14	
Mean(SD)	12.44 (5.09)	15.67 (6.66)	15.61 (6.68)	14.54 (6.35)	
ITest^†^ **					
Range	(0–10)	(0–10)	(0–10)	(0–10)	<0.0001
Median	5	5	4	5	
Mean(SD)	5.04 (1.65)	4.81 (1.73)	4.22 (1.76)	4.69 (1.75)	
DTest^†^ **					
Range	(0–10)	(0–10)	(0–10)	(0–10)	<0.0001
Median	3	3	3	3	
Mean(SD)	3.46 (1.86)	3.32 (1.91)	2.75 (1.89)	3.17 (1.91)	
QoL^†^ **					
Range	(15–48)	(12–48)	(12–48)	(12–48)	<0.0001
Median	32	34	37	34	
Mean(SD)	31.69 (5.58)	34.13 (6.23)	35.95 (6.26)	33.9 (6.28)	
Global activity limitation *					<0.0001
Limited	1257 (28%)	1567 (38%)	1535 (36%)	4359 (34%)	
Not limited	3156 (72%)	2543 (62%)	2773 (64%)	8472 (66%)	
SPH *					<0.0001
Poor	295 (7%)	334 (8%)	462 (11%)	1091 (9%)	
Fair	1043 (24%)	1248 (30%)	1261 (29%)	3552 (28%)	
Good	1568 (36%)	1570 (38%)	1766 (41%)	4904 (38%)	
Very good	1212 (27%)	669 (16%)	671 (16%)	2552 (20%)	
Excellent	295 (7%)	289 (7%)	148 (3%)	732 (6%)	

^†^ BMI=Body Mass Index, NCDs=Non-communicable diseases, ITest=Immediate verbal recall test, DTest=Delayed verbal recall test, QoL=Quality of Life, SPH=Self-perceived health. ^§^ Results in rows are range, median, and mean (SD) for quantitative indicators and counts (%) for qualitative indicators. NS means not statistically significant. * The Chi-squared test was used for categorical variables, ** the ANOVA test for continuous variables.

**Table 2:** Posterior generalized linear models (GLM) estimates of direct effects, with 95% credibility intervals, resulting from additive Bayesian networks (ABN) models in Italy, Spain, and Greece, 2015 year.

NCDs^†^	Italy ^§^	Greece ^§^	Spain ^§^
	β [95%CI]	β [95%CI]	β [95%CI]
BMI^†^	0.13 [0.11, 0.16]	0.09 [0.07, 0.12]	0.11 [0.08, 0.13]
Years of education	−0.07 [−0.09, −0.05]	−0.12 [−0.14, −0.09]	
Age (≥65 vs. <65)	0.12 [0.09, 0.14]	0.16 [0.14, 0.17]	0.08 [0.06, 0.10]
Living in couple (Not in couple vs. in couple)		0.11 [0.04, 0.17]	
Current job status (not employed vs. employed)			0.45 [0.37, 0.53]
SPH^†^	Italy	Greece	Spain
	β [95%CI]	β [95%CI]	β [95%CI]
NCDs^†^	−0.52 [−0.59, −0.44]	−0.57 [−0.64, −0.50]	−0.53 [−0.60, −0.46]
ITest^†^	0.07 [0.04, 0.11]	0.08 [0.04, 0.12]	0.07 [0.03, 0.10]
Dtest^†^	0.29 [0.26, 0.34]	0.16 [0.11, 0.21]	0.18 [0.14, 0.23]
Fluency	0.04 [0.02, 0.06]	0.08 [0.06, 0.11]	0.14 [0.12, 0.16]
Global activity limitations (Not limited vs. Limited)	1.99 [1.83, 2.16]	2.20 [2.03, 2.40]	2.02 [1.86, 2.21]
QoL^†^	0.12 [0.10, 0.14]		0.16 [0.15, 0.18]
Living in couple (Not in couple vs. in couple)	−0.36 [−0.53, −0.20]	−0.50 [−0.71, −0.30]	
Current job status (not employed vs. employed)	−0.65 [−0.83, −0.47]	−0.84 [−1.08, −0.64]	−0.39 [−0.61, −0.18]
Physical inactivity (Yes vs. No)	−0.63 [−0.81, −0.46]	−1.11 [−1.43, −0.85]	−0.85 [−1.06, −0.65]
Gender (Male vs. Female)			0.39 [0.23, 0.54]
Years of education			0.19 [0.12, 0.25]
QoL^†^	Italy	Greece	Spain
	β [95%CI]	β [95%CI]	β [95%CI]
Global activity limitations (Not limited vs. Limited)	0.08 [0.06, 0.09]	0.07 [0.05, 0.09]	0.08 [0.06, 0.09]
Household Total net income	0.03 [0.02, 0.04]	0.02 [0.01, 0.03]	0.02 [0.02, 0.04]
Household net worth coeff	0.02 [0.01, 0.03]	0.03 [0.02, 0.04]	0.03 [0.01, 0.03]
Gender (Male vs. Female)	0.04 [0.03, 0.05]		
Physical inactivity (Yes vs. No)	−0.05 [−0.06, −0.03]	−0.12 [−0.15, −0.09]	−0.08 [−0.09, −0.06]
SPH^†^ (Good vs. Less than good)		0.18 [0.16, 0.20]	
BMI^†^		−0.02 [−0.02, −0.01]	
NCDs^†^		−0.02 [−0.02, −0.01]	−0.02 [−0.02, −0.01]
ITest^†^		0.02 [0.01, 0.02]	0.03 [0.02, 0.03]
Current job status (not employed vs. employed)		−0.05 [−0.07, −0.03]	
Living in couple (Not in couple vs. in couple)			−0.03 [−0.04, −0.01]

^†^ NCDs = Non-communicable diseases, SPH = Self-perceived health, BMI = Body Mass Index, ITest = Immediate verbal recall test, DTest = Delayed verbal recall test, QoL = Quality of Life. A cell is empty when there is not an edge in the corresponding directed acyclic graphs (DAG).

## Supplementary Materials

The following are available online at https://www.mdpi.com/1660-4601/17/7/2414/s1, Table S1: Correlation Matrix between pairs of variables.
